# Species Radiation of Carabid Beetles (Broscini: *Mecodema*) in New Zealand

**DOI:** 10.1371/journal.pone.0086185

**Published:** 2014-01-23

**Authors:** Julia Goldberg, Michael Knapp, Rowan M. Emberson, J. Ian Townsend, Steven A. Trewick

**Affiliations:** 1 Department of Morphology, Systematics and Evolutionary Biology, J.F. Blumenbach Institute of Zoology & Anthropology, Georg-August-University Göttingen, Göttingen, Germany; 2 Department of Anatomy, University of Otago, Dunedin, New Zealand; 3 Department of Ecology, Lincoln University, Canterbury, New Zealand; 4 Independent Researcher, Levin, New Zealand; 5 Ecology Group, Institute of Agriculture and Environment, Massey University, Palmerston North, New Zealand; Australian Museum, Australia

## Abstract

New Zealand biodiversity has often been viewed as Gondwanan in origin and age, but it is increasingly apparent from molecular studies that diversification, and in many cases origination of lineages, postdate the break-up of Gondwanaland. Relatively few studies of New Zealand animal species radiations have as yet been reported, and here we consider the species-rich genus of carabid beetles, *Mecodema*. Constrained stratigraphic information (emergence of the Chatham Islands) and a substitution rate for Coleoptera were separately used to calibrate Bayesian relaxed molecular clock date estimates for diversification of *Mecodema*. The inferred timings indicate radiation of these beetles no earlier than the mid-Miocene with most divergences being younger, dating to the Plio-Pleistocene. A shallow age for the radiation along with a complex spatial distribution of these taxa involving many instances of sympatry implicates recent ecological speciation rather than a simplistic allopatric model. This emphasises the youthful and dynamic nature of New Zealand evolution that will be further elucidated with detailed ecological and population genetic analyses.

## Introduction

Biologists have long been perplexed with New Zealand's biotic composition which cannot be classed as typically oceanic or continental [1], [2]. The landmass is continental in stratigraphic composition and origin, founded on a tectonic fragment separated from Gondwanaland ∼80 million years ago (Ma) - referred to as Zealandia [3], [4], [5]. Its enigmatic biota includes so-called relicts (tuatara *Sphenodon*), a supposed Gondwanan element (e.g. leiopelmatid frogs, Onychophora, kauri *Agathis*) and recent colonists [6], [7], [8], [9], [10]. This eclectic biotic composition has singled New Zealand out as being a particularly intriguing example of island biology [11].

An initial emphasis by phylogeographers on the origin of New Zealand lineages (stem groups) tended to undervalue the greater evolutionary significance of crown groups in the assembly of the New Zealand biota. Increasingly, molecular studies are revealing the story of diversification in a wide variety of New Zealand animals and plants (see for example [10], [12], [13], [14], [15], [16], [17], [18], [19]). There appears to be little congruence in phylogeographic patterns expressed by New Zealand taxa with no overwhelming pattern among lineage formation, landscape history and distribution of taxa (see reviews in [8], [9], [20]). In retrospect this may not be surprising, for the early expectation that species level phylogeography would reveal scaled-down patterns of the type seen in the northern hemisphere have rarely been realised (but see [21], [22], [23]). On the contrary, even within species, genetic diversity is often very high [24] and spatial patterns often complex, indicating that even on the relatively small scale of New Zealand population diversity relates to older events and/or involves persistence of larger populations than those commonly identified for European taxa [18], [20].

In many respects northern hemisphere biology has been dominated by Pleistocene climate extremes causing fluctuating population size, but New Zealand biology appears to have an older signature and identification of the respective roles of past geophysical events is in its infancy [but see for example tiger beetles- [19], weta- [24], cicadas- [25]). We know that after separation from Gondwanaland starting 80 Ma, the continent of Zealandia gradually submerged beneath the sea, and that modern New Zealand is primarily the product of tectonic activity initiated ∼25 Ma [3], [4], [5]. How much land persisted in the region prior to this phase is debated, but extreme reduction is certain and this period is likely to have caused a biological bottleneck at the very least [26]. Thus, whatever the source of lineages in New Zealand (oversea dispersal or Zealandian survivors), crown group diversity is unlikely to be older than Miocene. Tectonic activity since that time has resulted in substantial remodelling of the archipelago, including crustal uplift and orogenesis since Pliocene time (∼5 Ma; [27], [28]). Biotic turnover since early Miocene time is also evident from the fossil record and this might reflect a shift from tropical to temperate climate (e.g. [29], [30], [31]). There is naturally an increasing interest in how diversification is distributed through time and space.

In this study we examine the phylogenetic relationships and timing of radiation of *Mecodema* (Blanchard, 1843) carabid beetles (tribe Broscini). This endemic genus of large, flightless beetles constitutes a prominent species radiation in New Zealand and presents a good opportunity to explore species level diversification. We utilise the fact that the genus is represented on the Chatham Islands, which are located approximately 850 km east of the South Island, New Zealand in the Pacific Ocean. Geological evidence for the formation of this archipelago within the last 4 Myr is compelling [3], [32], [33], [34] and corroborated by genetic data for many taxa (e.g. insects – [18], [35], plants – [36], [37], [38], parakeets – [39], pigeons – [40], [41], cicadas – [42], invertebrates and plants – [43]). In this study the earliest possible establishment of an island biota (4 Myr) on the Chathams [32] is used as a maximum possible calibration for estimating the timing of diversification in *Mecodema*. Furthermore a substitution rate for Coleoptera [44] is employed to further explore timing of lineage diversification of this beetle genus.

## Materials and Methods

### Sampling

The genus *Mecodema* (Blanchard, 1853) belongs to the tribe Broscini (Carabidae). Broscini has a worldwide distribution but has its main diversity in the southern hemisphere (subfamily Nothobroscinae) [45] and consists of at least 27 genera, comprising about 80 species (see http://www.landcareresearch.co.nz/research/biosystematics/invertebrates/carabid/carabidlist) [46], [47]. Six endemic genera of Broscini are recognized in New Zealand, but *Mecodema* is especially species-rich. Adult *Mecodema* beetles are relatively slow-moving, nocturnal, flightless (with fused elytra), generally active throughout the year, and usually scarce [48]. As with other Carabidae, adults and larvae of the New Zealand taxa are predatory. *Mecodema* is a diverse genus with species distributed throughout the New Zealand mainland from alpine to coastal habitats. In contrast, there is a single species (*Mecodema alternans*) on the Chatham Islands. The same species occurs in southeast New Zealand near Dunedin. Although *M. alternans* may be better treated as a species complex [47], no morphological characters have yet been described that distinguish Chatham Island populations from those in mainland New Zealand ([49] & I. Townsend pers. obs.).

In total our sampling comprised 113 specimens, with 88 *Mecodema* representing 35 described species, and 4 undescribed species of the 66 recognized *Mecodema* species (after [45], [46] and http://www.landcareresearch.co.nz/research/biosystematics/invertebrates/carabid/carabidlist), see Table 1 for details and authorities. Putative outgroup New Zealand Broscini in our sample included *Oregus* (Putzeys, 1868), *Diglymma* (Sharp, 1886), *Brullea antarctica* (Laporte de Castelnau, 1867), *Metaglymma* (Bates, 1867), and *Bountya insularis* (Townsend, 1971); plus one representative of Broscini from Australia, *Chylnus ater* (Putzeys, 1868) (Table 2). As many of the species in *Mecodema* are scarce and therefore difficult to collect we also made use of material from museum collections, and supplemented outgroup sampling with available GenBank sequences (*Oregus septentrionalis* AF466847 & AF466848; *Oregus crypticus* AF54423; *Oregus inaequalis* AF466850 [50]; *Calathus aztec* GU254333 [51]; *Broscosoma relictum* AF012502; *Promecoderus* sp. AF012499; *Creobius eydouxi* AF012498 [52]). The resulting sample represents the geographic and ecological range of *Mecodema* in New Zealand (Table 2). *Mecodema* species show examples of allopatric, parapatric and sympatric distribution. Species but not always species groups are limited to particular areas and only few species (e.g. *M. crenicolle*) are documented as being present in both main islands of New Zealand [47].

**Table 1:** List of Broscini species used in this study with information regarding authority, distribution (S.I. = South Island New Zealand, N.I. = North Island New Zealand; Ch.Is. = Chatham Islands) and number of individuals employed.

Species	Distribution	# used in study	Authority
*M. alternans*	S.I. & Ch.Is.	12	Laporte de Castelnau, 1867
*M. alternans hudsoni*	The Snares	1	Broun, 1909
*M. crenicolle*	N.I. & S.I.	9	Laporte de Castelnau, 1867
*M. crenaticolle*	N.I.	5	Redtenbacher, 1868
*M. curvidens*	N.I.	1	(Broun, 1915)
*M. fulgidum* (cf *fulgidum*)	S.I.	5	Broun, 1881
*M. howittii*	S.I.	2	Laporte de Castelnau, 1867
*M. longicolle*	N.I.	1	Broun, 1923
*M. lucidum*	S.I.	2	Laporte de Castelnau, 1867
*M. occiputale*	N.I.	3	Broun, 1923
*M.* cf *oconnori*	N.I.	4	Broun, 1912
*M. oregoides*	S.I.	2	(Broun, 1894)
*M. rugiceps*	S.I.	1	Sharp, 1886
*M. sculpturatum*	S.I.	4	Blanchard, 1843
*M. huttense* (cf *huttense*)	S.I.	3	Broun, 1915
*M. simplex*	N.I.	4	Laporte de Castelnau, 1867
*M. spinifer*	N.I.	4	Broun, 1880
*M. strictum*	S.I.	1	Britton, 1949
*M. sulcatum*	N.I. & S.I.	1	(Sharp, 1886)
*M. validum*	N.I.	1	Broun, 1923
*M. rectolineatum*	S.I.	1	Laporte de Castelnau, 1867
*M. politanum*	S.I.	1	Broun, 1917
*M. impressum*	S.I.	1	Laporte de Castelnau, 1867
*M. constrictum*	S.I.	3	Broun, 1881
*M. costellum lewisi*	S.I.	1	Broun, 1908
*M. costellum obesum*	S.I.	1	Townsend, 1965
*M. allani*	S.I.	1	Fairburn, 1945
*M. laterale*	S.I.	1	Broun, 1917
*M. minax*	S.I.	2	Britton, 1949
*M. elongatum*	S.I.	1	Laporte de Castelnau, 1867
*M. metallicum*	S.I.	1	Sharp, 1886
*M. ducale*	S.I.	1	Sharp, 1886
*M. morio*	S.I.	1	(Laporte de Castelnau, 1867)
*M. infimate*	S.I.	1	Lewis, 1902
*M. punctatum*	S.I.	1	(Laporte de Castelnau, 1867)
*Meta. moniliferum*	S.I.	2	Bates, 1867
*Meta. aberrans*	S.I.	5	Putzeys, 1868
*Meta. tibiale*	S.I.	1	(Laporte de Castelnau, 1867)
*Brullea antarctica*	N.I. & S.I.	1	Laporte de Castelnau, 1867
*Bountya insularis*	Bounty Is.	1	Townsend, 1971
*O. aereus*	S.I.	6	(White, 1846)
*O. inaequalis*	S.I.	2	(Laporte de Castelnau, 1867)
*O. crypticus*	S.I.	1	Pawson, 2003
*O. septentrionalis*	S.I.	2	Pawson, 2003
*D. clivinoides*	S.I.	4	(Laporte de Castelnau, 1867)
*D. obtusum*	S.I.	2	(Broun, 1886)
*D. seclusum*	S.I.	1	(Johns, 2007)

**Table 2:** List of investigated Broscini samples with sample numbers corresponding to numbers in Figures, including species name, genes sequenced per specimen and sampling locations.

Sample ID	Species	Genes	Location
MB 01*	*M. alternans*	a	Chatham Is., South East Is.
MB 02*	*M. alternans*	b	Chatham Is., South East Is.
MB 70*	*M. alternans*	a	Chatham Is., Mangere Is.
MB 71*	*M. alternans*	b	Chatham Is., South East Is.
MB 86*	*M. alternans*	b	Chatham Is., Mangere Is.
MB 87*	*M. alternans*	b	Chatham Is., South East Is.
MB 86.1*	*M. alternans*	d	Chatham Is., Mangere Is
MB 88*	*M. alternans*	d	Chatham Is., South East Is.
MB 88.1*	*M. alternans*	d	Chatham Is., South East Is.
MB 190*	*M. alternans*	d	S.I., Otago, Takahopa River Mouth
MB 14*	*M. alternans*	a	S.I., Dunedin, Taieri Mouth
MB 16*	*M. alternans*	a	S.I., Dunedin, Sandfly Bay
MB185*	*M. alternans hudsoni*	d	The Snares [LUNZ2804]
MB 61*	*M. crenaticolle*	d	N.I., Taranaki, Lake Rotokare
MB 62*	*M. crenaticolle*	c	N.I., Taranaki, Lake Rotokare
MB 72*	*M. crenaticolle*	d	N.I., Tongariro, Ohakune
MB 12*	*M. crenicolle*	d	S.I., Nelson, Shenandoah
MB 44*	*M. crenicolle*	d	S.I., Abel Tasman, Awaroa
MB 82*	*M. crenicolle*	d	S.I., Abel Tasman, Awaroa
MB 79*	*M. crenicolle*	b	S.I., Marlborough Sounds, Pelorus Bridge
MB 103*	*M. crenicolle*	b	S.I., Nelson Lakes, Wairau River
MB 117*	*M. crenicolle*	d	S.I., Lake Rotoroa, Braeburn Walk
MB 163*	*M. crenaticolle*	c	N.I., Taranaki, Kaiteke Ra., Lucy's Gully
MB 186*	*M. crenaticolle*	d	N.I., Waikato, Skyline Cave
MB 54*	*M. morio*	d	S.I., Catlins, Purakaunui Falls
MB 105*	*M. infimate*	c	S.I., Codfish Island
MB 110*	*M. fulgidum*	a	S.I., Clarence Valley, Mt. Percival
MB 91*	*M.* cf *fulgidum*	b	S.I., Seaward Kaikoura Ra., Mt. Lyford
MB 123*	*M. fulgidum*	d	S.I., Hamner Springs, Forest Park
MB 81*	*M. fulgidum*	d	S.I., Cobb Valley, Lake Sylvester Tr.
MB 07*	*M. fulgidum.*	d	S.I., Seaward Kaikoura Ra., Mt. Fyffe
MB 17*	*M. punctatum*	d	S.I., Rock & Pillar Range
MB 98*	*M. howittii*	a	S.I., Canterbury, Banks Peninsula
MB 99*	*M. howittii*	b	S.I., Canterbury, Banks Peninsula
MB 50*	*M. spinifer*	b	N.I., Hawkes Bay, Mohi Bush
MB 18*	*M. spinifer*	a	N.I., Auckland, Waitakeres, Arataki
MB 10*	*M. spinifer*	d	N.I., Auckland, Waitakeres Ra.
MB 50.1*	*M. spinifer*	d	N.I., Hawkes Bay, Mohi Bush
MB 100a*	*M. costellum lewisi*	c	S.I., Road nr. Mt. White Station
MB 101*	*M. costellum obesum*	d	S.I., Nelson, Takaka Hill, Canaan
MB 195*	*M. allani*	d	S.I., Nelson Lakes, Matakitaki V. [LUNZ2372]
MB 197*	*M. laterale*	d	S. I., Fiordland, Routeburn Track [LUNZ2383]
MB 178	*M. minax*	d	S.I., Catlins, Mt. Pye Summit [P23]
MB 180*	*M. minax*	c	S.I., Catlins, Mt. Pye Summit [P22]
MB 160*	*M. elongatum*	c	S.I., Otago, Kinloch
MB 143*	*M. metallicum*	c	S.I., Buller, Fox River
MB 176*	*M. rectolineatum*	d	S.I., Remarkables Range, Wye Creek [P15]
MB 196*	*M. politanum*	d	S.I., The Remarkables, Rastus Burn [LUNZ2640]
MB 128*	*M. impressum*	d	S.I., Queenstown, Kinloch
MB 134*	*M. constrictum*	d	S.I., Craigieburn Forest P., Education Ct.
MB 35*	*M. constrictum*	c	S.I., Fog Peak, Porter's Pass
MB 27*	*M. constrictum*	d	S.I., Canterbury, Craigieburn Rec. area
MB 121*	*M. ducale*	c	S.I., Lewis Pass, Lake Daniels Walk
MB 20*	*M.* cf *oconnori*	d	N.I., Levin, 30B The Avenue
MB 73*	*M.* cf *oconnori*	c	N.I., Te Urewera, Ngamoko Trig Tr.
MB75*	*M.* cf *oconnori*	d	N.I., Dannevirke, Norsewood Res.
MB 21*	*M.* cf *oconnori*	a	N.I., Wellington, Levin, Ohou
MB 76*	*M. simplex*	d	N.I., Tararua Forest Park, Putara
MB 77*	*M. simplex*	c	N.I., Tararua Ra., Mt Holdsworth
MB 25*	*M. simplex*	b	N.I., Manawatu, Pahiatua Track
MB 64*	*M. simplex*	a	N.I., Manawatu, Palmerston North
MB 63*	*M. longicolle*	a	N.I., Ruahine Ra., Pohangina Valley
MB 69*	*M. validum*	a	N.I., Tongariro NP, Whakapapanui Track
MB 90*	*M. oregoides*	a	S.I., Christchurch, Ahuriri Scenic Res.
MB 147.1*	*M. oregoides*	c	S.I., Christchurch, Ahuriri Scenic Res.
MB 19*	*M. lucidum*	a	S.I., Otago, Carrick Range
MB 111*	*M. lucidum*	d	S.I., Pisa Range
MB 03*	*M. rugiceps*	a	S.I., Fiordland, Lake Harris
MB 45*	*M. sculpturatum*	a	S.I., Dunedin, Ross Reserve
MB 125*	*M. sculpturatum*	d	S.I., Catlins Forest Park, River Walk
MB 04*	*M. sculpturatum*	d	S.I., Dunedin, Leith Saddle
MB 06*	*M. sculpturatum*	d	S.I., Dunedin, Mosgeil, Silver St.
MB 09*	*M. huttense*	c	S.I., Canterbury, Peel Forest
MB 46*	*M.* cf *huttemse*	d	S.I., Canterbury, Peel Forest
MB 108*	*M.* cf *huttense*	b	S.I., Canterbury, Peel Forest
MB 96*	*M. strictum*	a	S.I., Nelson, Takaka Hill, Canaan
MB 95*	*M. sulcatum*	a	S.I., Kaikoura, North of Ohau Point
MB 66*	*M. curvidens*	a	N.I., BOP, Rotorua
MB 68*	*M. occiputale*	a	N.I., Mangatoi, Otanewainuku Forest
MB 65*	*M. occiputale*	d	N.I., BOP, Ohope Scenic Res.
MB 67*	*M. occiputale*	d	N.I., Mangatoi, Otanewainuku Forest,
MB 11*	*M.* n.sp.	a	S.I., Central Otago, Old Man Range
MB 37*	*M.* n.sp.	b	S.I., Arthurs Pass NP, Dome
MB 51.1*	*M.* n.sp.	a	N.I., Hawkes Bay, Havelock North
MB 124*	*M*. n.sp.	c	S.I., Lewis Pass, Lewis Tops
MB 30*	*M. crenicolle*	d	S.I., Abel Tasman, Awaroa
MB 38*	*M. crenicolle*	d	S.I., Takaka, The Grove
MB 49*	*M. crenicolle*	d	S.I., Takaka, Rameka Track
MB 106	*Meta. moniliferum*	a	S.I., Canterbury, Christchurch, Quail Is.
MB 107	*Meta. moniliferum*	b	S.I., Canterbury, Christchurch, Quail Is.
MB 198	*Meta. aberrans*	d	S.I., Canterbury, Lake Tekapo [LUNZ3089]
MB 187*	*Meta. aberrans*	d	S.I., Otago, Cromwell, Bendigo
MB 181	*Meta. aberrans*	d	S.I., Otago, Old Man Range, Omeo Gully [P16]
MB 182	*Meta. aberrans*	d	S.I., Otago, Old Man Range, Omeo Gully [P17]
MB 184	*Meta. aberrans*	d	S.I., Otago, Old Man Range, Omeo Gully [P10]
MB 194	*Meta. tibiale*	d	S.I., Central Otago, Upper Clutha [LUNZ3088]
MB 85	*Brullea antarctica*	c	N.I., Manawatu, Himatangi Beach
MB 199	*Bountya insularis*	d	Bounty Is., Proclamation Is.
MB 192	*Chylnus ater*	d	Australia
MB 13	*O. aereus*	b	S.I., Otago, Danseys Pass
MB 41	*O. aereus*	a	S.I., Dunedin, Morrison St.
MB 28	*O. aereus*	d	S.I., Dunedin, 46 Morrison St.
MB 29	*O. aereus*	d	S.I., Dunedin, Sandfly Bay
MB 47	*O. aereus*	d	S.I., Dunedin, Silver Peaks
MB53	*O. aereus*	d	S.I., L. Onslow, Lammarlaws
MB 5	*O. inaequalis*	d	S.I., Dunedin, Miller Rd.
MB 48	*D. clivinoides*	b	S.I., Seaward Kaikoura Ra., Tinline Va.
MB 31	*D. clivinoides*	a	S.I., NW Nelson, Heaphy Track
MB 161	*D. obtusum*	d	S.I., Fiordland, Kepler Track
MB 162	*D. obtusum*	c	S.I., Otago, Catlins Coast, Tautuku
MB 159	*D. clivinoides*	c	S.I., Otago, Kinloch
MB 8	*D. clivinoides*	d	S.I., Nelson, Cobb Valley
MB 175	*D. seclusum*	d	S.I., Fiordland, Spey River Valley

(*M.* = *Mecodema*; *Meta* = *Metaglymma*; *O.* = *Oregus*; *D.* = *Diglymma*; S.I. = South Island New Zealand; N.I. = North Island New Zealand; BOP = Bay of Plenty; genes analysed: a = COI, COII, 16S, 18S; b = COI, COII, 16S; c = COI, 18S; d = COI; LUNZ = Lincoln University Entomological Research Museum; P = S.M. Pawson collection);

COI sequences from samples of the *Mecodema* ingroup marked with * have previously been deposited in Genbank (#JN409817–JN409904) [18].

Fresh specimens were obtained in accordance with Department of Conservation collection permits and preserved in 95% ethanol after hand collection in the field. These specimens are in interim storage with unique voucher numbers as part of the Phoenix Collection, Massey University, Palmerston North. Additional taxa were loaned from relevant collections (labelled accordingly in Table 2). Species identification relied on morphological characters [46].

### DNA extraction, amplification and sequencing

DNA was extracted from a single leg of each collected specimen using a salting-out extraction protocol [53] or a CTAB and phenol/chloroform extraction [54]. DNA extractions from pinned museum specimens were undertaken in a dedicated ancient DNA laboratory, remote from modern DNA facilities, using a CTAB and phenol/chloroform extraction [54] and following stringent protocols for handling ancient DNA [55]. We employed three mitochondrial genes and one nuclear for determining the species level relationships in *Mecodema*. Partial cytochrome oxidase I (COI) fragment was amplified for 24 specimens in this study and the remaining 89 COI sequences were drawn from GenBank (JN JN409817–JN409904), see Table 2. Additionally, partial cytochrome oxidase II (COII) and 16S were amplified for a subset of 39 specimens. The COI gene region (789 bp) was amplified using primers C1-J-2195 and L2-N-3014, COII (589 bp) using primers TL2-J-3037 and C2-N-3661, and 16S (769 bp) using N1-J-12585 and LR-N-13398 [56]. For museum specimens, which were expected to yield fragmented, low concentration DNA, *Mecodema*-specific COI primers were designed using the program Oligo4 (Molecular Biology Insights, Inc., Cascade, CO) to generate a series of short (∼130–200 bp) overlapping fragments (Table 3). Additionally a 967 bp fragment of nuclear rRNA (18S) for a subset of 44 Broscini was obtained using primers 18S-S22 and 18S-A1984 [57].

**Table 3:** List of *Mecodema* specific oligonucleotide primers for mtDNA cytochrome oxidase I (COI), designed for this study.

Primer name	Sequence
MB_COI38	^3′^ GCTGATGTAAAATATGCTCG
MB_COI39	^5′^ GAGCATATTTTACATCAGCAAC
MB_COI03	^3′^ CAATGAATAAATCCTCCAA
MB_COI05	^5′^ GGAGGATTTATTCATTGAT
MB_COI91	^3′^ GTWGATCCAATTGATGAAAC
MB_COI88	^5′^ GTARTTTCATCAATTGGATC
MB_COI12	^3′^ CTTAAATATGATCATGTRG

Polymerase chain reactions were performed in 10 µl volumes and the amplified products then checked on a 1% agarose gel and purified using SAP/EXO1 digest (USB Corporation). Purified PCR products were sequenced using standard protocols for the ABI Prism BigDye Terminator Ready Reaction Kit (Applied Biosystems, Mulgrave, Australia) and run on an ABI Prism 377 automated sequencer (Applied Biosystems). Sequence identity was confirmed by comparison with published data, checked for nucleotide ambiguities in Sequencher 4.2 (Gene Codes Corporation, Ann Arbor, MI, www.genecodes.com) and aligned using Se-Al v2.0a11 [58]. The sequences have been deposited with accession numbers KF913050–913193 at GenBank (16S: KF913050–913088; 18S: KF913089–913130; COII: KF913131–913169; COI: KF913170–913193).

### Phylogenetic analysis

To test whether *Diglymma* and *Oregus* species are the natural sister group to *Mecodema* we first analysed data from two genes separately (COI and 18S) as it was not possible to gain sequences for outgroup taxa outside of New Zealand for all the employed species and sequence availability in GenBank within Broscini was also very poor. Although *Oregus* and *Diglymma* represent two of the New Zealand Nothobroscina genera considered closest to *Mecodema* [45], it was crucial to verify this relationship within Broscini as two other potential outgroup taxa, *Metaglymma* and *Brullea*, exist. MrBayes 3.1.2 [59] was used to implement Bayesian analysis with the datasets applying a GTR model with gamma-distributed rate variation across sites and a proportion of invariable sites. Analyses with MrBayes used four independent Markov Chain Monte Carlo (MCMC) runs for ten million generations with a burn-in of 10% and a tree sampling frequency of 1000. Results were checked for convergence. Resulting posterior probabilities on the nodes were recorded.

To examine the species phylogeny of the *Mecodema* group in New Zealand we employed all four genes (three mitochondrial and one nuclear) with a subset of 50 taxa (44 ingroup and 6 outgroup samples). The outgroup sampling was chosen after consideration of the results from the prior outgroup analyses. All taxa with data missing for no more than one of four genes were included in the phylogenetic analysis (Table 2). Partition-homogeneity tests (PHT [60]) were implemented in PAUP*4.0b10 [61] with 500 replicates for the combination of the gene regions to detect significant heterogeneity among the data sets.

MrBayes 3.1.2 [59] was then used to implement Bayesian analysis with the concatenated dataset, applying a GTR model with gamma-distributed rate variation across sites and a proportion of invariable sites. The same model was applied to the partitions with rates and nucleotide frequencies for each gene unlinked. Analyses with MrBayes used four independent Markov Chain Monte Carlo (MCMC) runs for two million generations with a burn-in of 25% and a tree sampling frequency of 1000. Resulting posterior probabilities on the nodes were recorded. The same data were subjected to Maximum Likelihood analysis with bootstrap resampling incorporating a GTR model with gamma-distributed rate variation. ML analysis used RaxML [62] implemented via the CIPRES portal [63]. The data were partitioned by gene (COI, COII, 16S, 18S) and bootstrap resampling was halted by RaxML

### Molecular dating

As fossil remains of *Mecodema* that could provide information for calibrating a molecular clock have not been found, yet, we had to rely on geological information and a substitution rate calculated for Coleoptera COI [44] for calibration. In order to gauge the timing and extent of species radiation in *Mecodema* within New Zealand COI sequences were obtained for additional specimens in addition to previous analyses (Table 2). In some cases, this drew upon museum specimens to further assess the stability of our inferences about the distribution of diversity and timing of radiation in this beetle group.

In total 113 unique COI sequences were used for molecular dating in *Mecodema* (Table 2). To obtain estimates for the maximum age of lineage formation within the genus *Mecodema* we used this dataset of COI with two different calibration strategies. First we employed a stratigraphic calibration to estimate divergence times using the split between Chatham Island *M. alternans* and its closest relative on mainland New Zealand. We assumed a normal distribution for the age around a calibration value of 4 Myr, derived from the maximum age for the Chatham Islands land surface [3], [33], assuming that colonization was most likely sooner after emergence of the islands than later. Alternatively, to capture the minimum likely diversification dates we also calibrated the COI dataset with the substitution rate estimated by Pons et al. [44] for Coleoptera COI. This included a normally distributed prior on the substitution rate of 0.08606 subst/site/myrs/l, and a 95% HPD interval from 0.0253–0.147 subst/site/myrs/l as an approximation to the posterior distribution provided by Pons et al. [44]. This rate obtained from analysis of numerous beetle taxa is amongst the highest estimated for any animal gene, and other rates obtained for particular beetle lineages are much slower (e.g. 0.0211 subst/site/myrs/l [64], 0.02 subst/site/myrs/l [65]). Even these rates are nearly twice the widely employed 0.0115 subst/site/myrs/l estimate of Brower [66]. Age estimation for both datasets and both calibration strategies were conducted under the assumption of a strict molecular clock as well as assuming a relaxed molecular clock with a lognormal distribution of rates along the phylogenies [67]. The fit of both priors was compared using Bayes Factors. For all datasets and calibration strategies the relaxed lognormal clock fitted the data decisively better than the strict clock [68].

We used the software BEAUTI 1.4.8 [69] and BEAST 1.7.5 [70] for molecular dating with the given calibrations. All analyses were conducted with a Birth-Death tree prior and a random starting tree under the GTR+I+Γ model of nucleotide substitution. The MCMC was run for 50–100 million generations, sampling every 5000^th^–10,000^th^ step after a discarded burn-in of 5–10 million steps. Each analysis was run at least two times. The program Tracer 1.4 [71] was used to summarize posterior distributions of all parameters in question, to verify convergence of the MCMC and to estimate Effective Sample Sizes (ESS). If the effective sample size was less than 200, a third MCMC run was conducted for the respective analysis. After convergence of the MCMC was confirmed, the posterior distributions of all parameters were estimated from the combined posterior distributions of all runs conducted for each analysis. The program FigTree 1.4.0 [69] was used to visualize the reconstructed phylogenies.

## Results

Three widely used mitochondrial gene regions were employed to gauge the scale of genetic diversity among the *Mecodema* specimens. Overall we observed relatively low genetic distances among species of *Mecodema* with a maximum ML-distance of 0.0179 in COII (COI: 0.0161, 16S: 0.053). Lower values for 16S compared to COI and COII reflect the comparatively low proportion of variable sites in this gene (16.9%).

**Figure 1:**
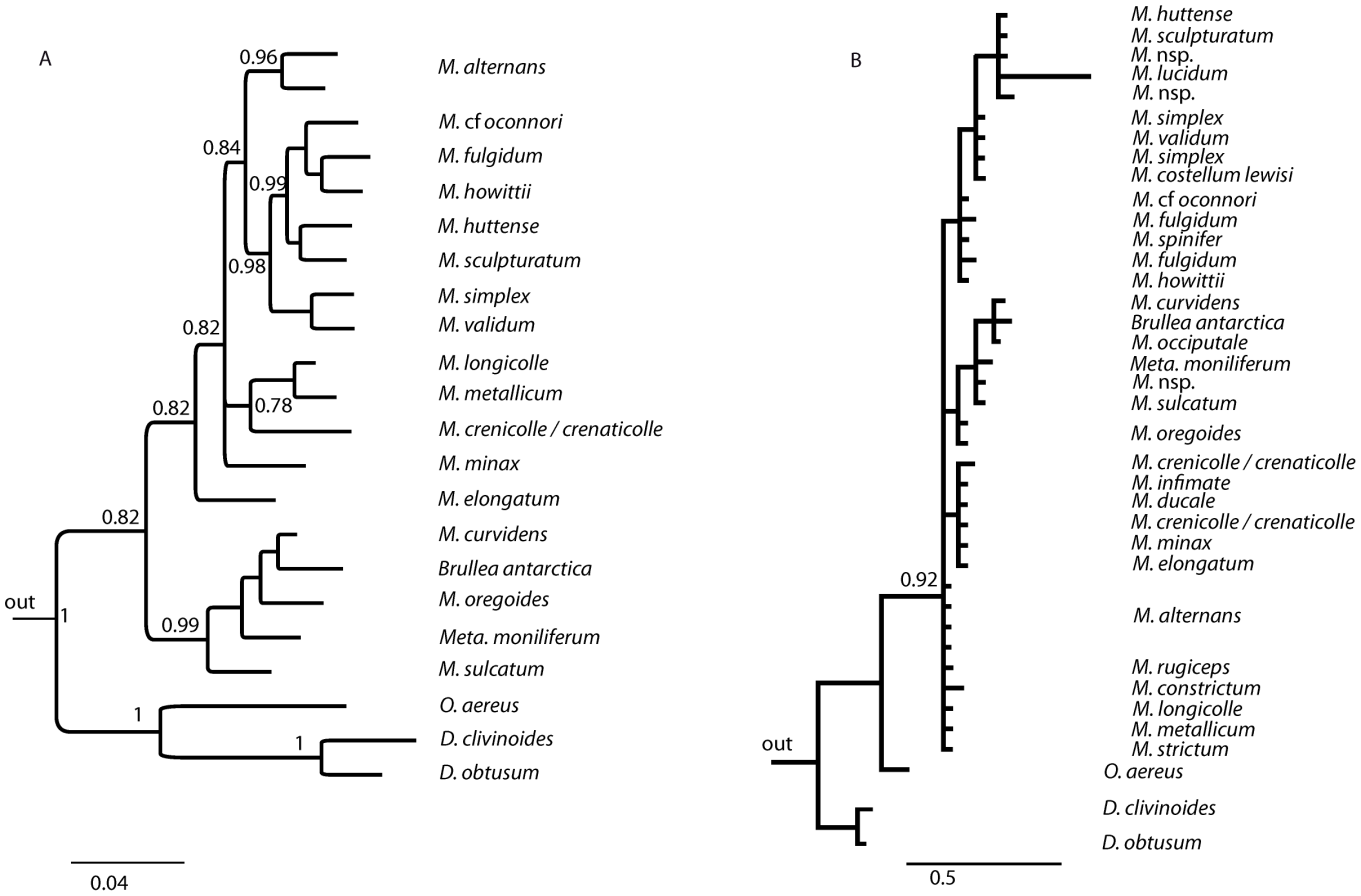
Bayesian phylogenetic trees of New Zealand Broscini. Bayesian trees of a subset of Broscini samples to confirm ingroup and outgroup relationship; A) COI phylogney (10 million generation, run four times, burn in 10%), three taxa outside New Zealand Broscini (*Bountya insularis*, *Chylnus ater*, *Calathus aztec*) were chosen as outgroup (not shown); B) 18S phylogeny (10 million generation, run four times, burn in 10%), three taxa outside New Zealand Broscini (*Broscosoma relictum*, *Promecoderus* sp., *Creobius eydouxi*) comprised the outgroup (not shown).

Separate analyses of COI and 18S DNA sequences from 41 specimens of *Mecodema* and 6 outgroup taxa (Table 2) resulted in similar topologies even though sequence variation in 18S was low. These analyses confirmed *Oregus* and *Diglymma* as the sister group to *Mecodema*, and revealed the placement of *Metaglymma* and *Brullea* within the *Mecodema* radiation (Figs. 1A & B).

**Figure 2:**
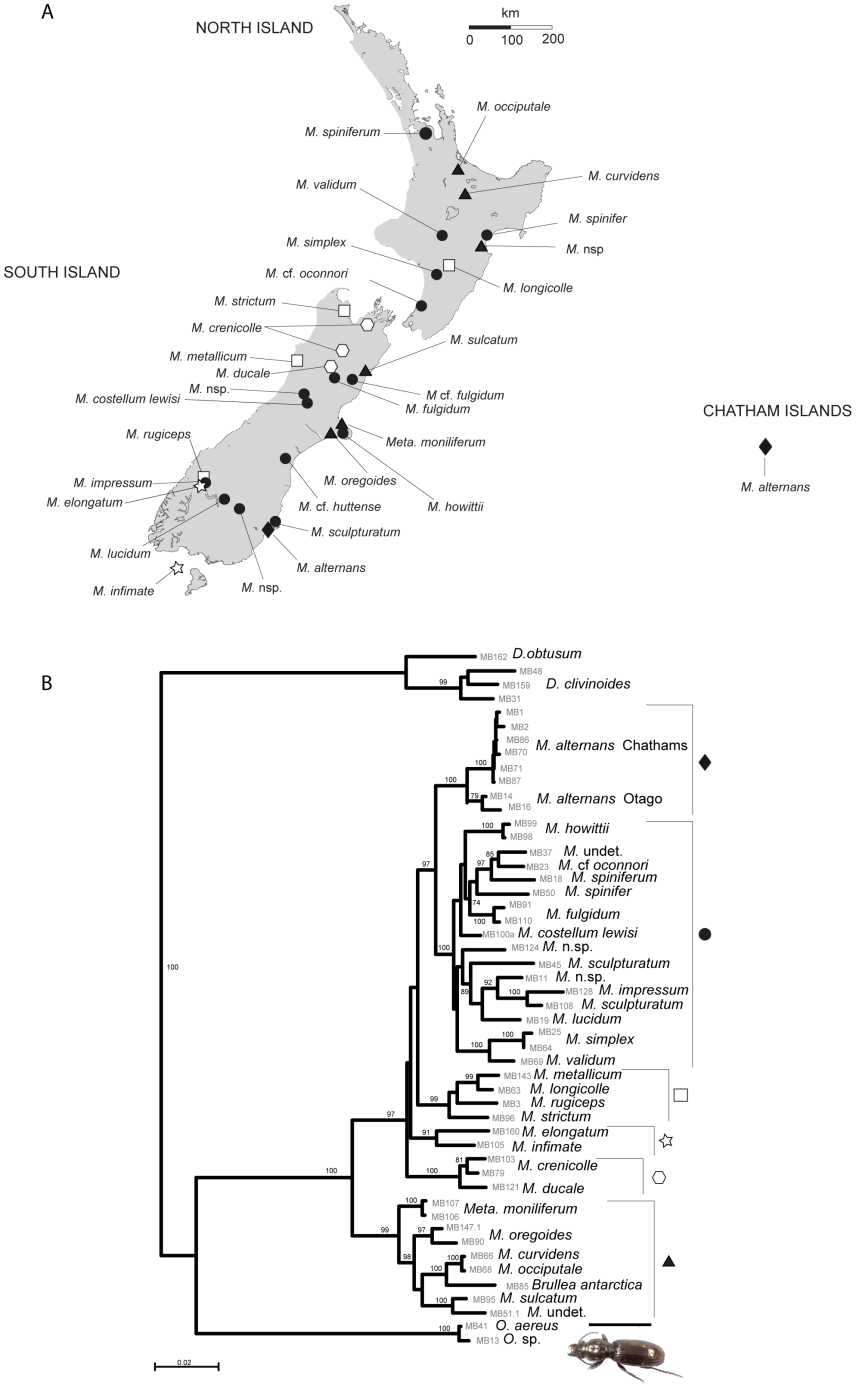
Maximum likelihood phylogeny for *Mecodema*. A) Spatial distribution of samples used in this analysis. Symbols correspond to those in Fig. 2B and code for different clades. B) Analysis of concatenated dataset including mitochondrial COI, COII and 16S plus nuclear 18S. Values at nodes indicate ML bootstrap support returned by analysis using RaxML. Specimen numbers at tips are given as in Table 2.

The alignment of data from four gene regions comprising 50 specimens sampled across New Zealand (including 6 outgroup specimens –Table 2, Fig. 2) was 3114 bp long in total. All three mitochondrial genes displayed the average insect A-T content of about 75%. The partition homogeneity test (PHT) revealed no significant heterogeneity of lineage partitioning among the data sets (*p* = 0.866), suggesting their concatenation was appropriate. The GTR+I+Γ model of nucleotide substitution was identified as the best fitting model by the hLRT and the AIC as implemented in Modeltest 3.5 [72].

Bayesian and ML analysis of the concatenated dataset supported a single topology with the same groupings and branching order and well-supported nodes (Fig. 2). *Mecodema* was confirmed as paraphyletic with respect to *Metaglymma* and *Brullea* as these fall inside the *Mecodema* complex throughout all datasets and analyses in this study. We note that in all cases, *Metaglymma* and *Brullea* are placed within the *M. curvidens/origoides* group. This phylogenetic position contradicts the current taxonomic classification and needs to be addressed further in the future. Clades revealed in this analysis comprise species that are, in many cases, widely distributed across New Zealand, and therefore spatial structure is not evident (Fig. 1A).

**Figure 3:**
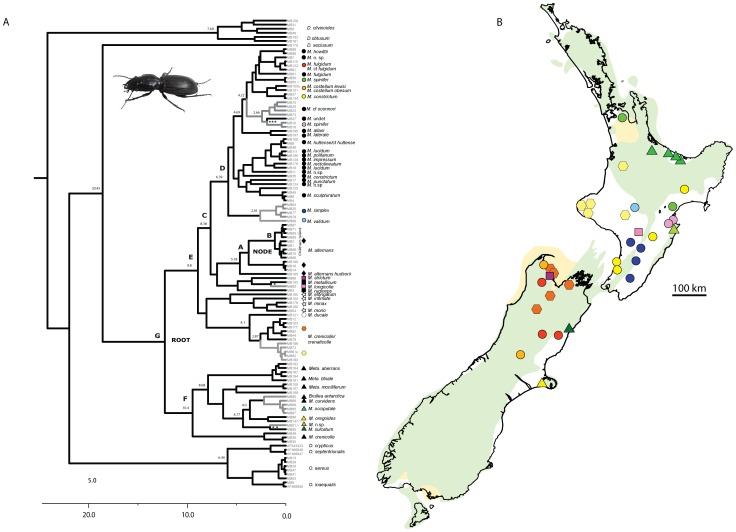
The timing of *Mecodema* diversification. A) COI Bayesian tree generated in BEAST. Numbers on nodes show age estimates based on stratigraphic calibration of 4 Myr. Outgroup taxa were the same as used in the outgroup test (Fig. 1). Grey branches indicate lineages present in North Island New Zealand, and black branches indicate South Island lineages. Tip symbols correspond to clade and location identifiers in Fig. 2. Coloured symbols match symbols in Fig. 3B, highlighting disjunct lineages in southern North Island and northern South Island. Asterisks indicate age estimates between N.I. and S.I. lineages (* = 1.60 Myr, ** = 1.69 Myr, *** = 2.03 Myr). B) Reconstruction of the paleogeographic environment in lower North Island, New Zealand ca. 3 million years ago, green areas indicating likely land above sea level during this time (modified from [28]). Black outlines indicate present day New Zealand land area with coloured symbols corresponding to those in Fig. 3A, showing the present sampling locations of some sister taxa in North Island and South Island. (Scale bar = 100 km.)

The complete alignment of the COI gene region comprising 113 specimens (including 20 outgroup specimens within Broscini and another one outside of this tribe) was 789 bp long and had an overall A-T bias of 73%. *Diglymma* and *Oregus* species that represent two of the New Zealand Nothobroscina genera taxonomically closest to *Mecodema* [45] grouped, as expected, outside of the *Mecodema* clade (Fig. 3). The other potential sister taxa to *Mecodema* within Broscini, *Metaglymma* and *Brullea*, were not supported as being sister to *Mecodema*, but were nested within the *Mecodema* radiation (Fig. 3). The relationship among *Mecodema* species was consistent with other analyses conducted in this study, with clades similar to species groupings previously proposed [47]. Despite a good level of resolution in the gene trees, there was little evidence for spatial correlation of clades with the current terrain of New Zealand. In keeping with our four gene analysis, it was clear that no groupings of species in specific North/South Island clades are apparent, nor are specific lineages correlated to landscapes (Figs. 2 & 3).

Accommodating a relaxed molecular clock approach we calculated divergence times by first using BEAST with a stratigraphic calibration and set priors for the NODE of 4 Myr (normal distribution), followed by use of a substitution rate estimated for COI in Coleoptera [44]. The two approaches resulted in different age estimates within the *Mecodema* radiation. For the given dataset of 113 taxa the ROOT was estimated at a maximum age of 13.42 Myr with the stratigraphic calibration of 4 Myr. However, using the rate of molecular evolution proposed from independent data for COI in Coleoptera [44], we found that date estimates were more than an order of magnitude smaller with 0.83 Myr for the ROOT and 0.27 Myr for the NODE (Table 4). Time estimates for principal nodes of the stratigraphic calculation are also shown in Fig. 3A.

**Table 4:** BEAST time estimates based on stratigraphic and COI substitution rate of Coleoptera [44] calibration.

Node	Estimates Myr (95% HPD) with stratigraphic calibration	Estimates Myr (95% HPD) with rate calibration
**A**	**4**	**0.27 (0.08–0.46)**
B	1.24 (0.5–2.09)	0.07 (0.02–0.15)
C	8.38 (5.29–12.06)	0.52 (0.22–0.9)
D	6.39 (3.97–9.41)	0.4 (0.18–0.72)
E	9.8 (5.86–13.94)	0.6 (0.27–1.07)
F	10.4 (5.86–15.93)	0.64 (0.26–1.12)
**G**	**13.42 (7.84–19.51)**	**0.83 (0.37–1.45)**

Letters correspond to letters in Fig. 3A, bold letters denote NODE (A) and ROOT (G).

Most of the *Mecodema* radiation appeared to be geologically young, with estimates of many clade origins less than 5 Myr. There was little spatial correlation within the COI dataset even at a broad scale such as between the two islands. Instead we noted multiple instances of North Island/South Island (NI/SI) splits within the phylogram (Fig. 3A, grey/black branches with coloured symbols correlating to map in Fig. 3B), at least one in each of the taxonomic groups in *Mecodema*. All but one of these splits (between the *simplex*/*validum* group to the rest of the inner clade) were younger than 5 Myr. The results estimated based on the substitution rate of Coleoptera [44] were even younger and placed the origin of all analysed *Mecodema* clades within the last 1 Myr (Tab. 4).

## Discussion

In this study we explored the pattern and depth of species diversity in the beetle genus *Mecodema*, which was sampled broadly across known species subgroups [47] and geographic range in mainland New Zealand, the Snares Islands and the Chatham Islands. Trees with well-supported structure were returned from the combined data set, although some shallow species-level relationships remain unresolved. Most clades are stable across analyses and are largely consistent with the taxonomic groupings proposed by [46] and [47]. Incomplete resolution of some species-level relationships in *Mecodema* reflects the low levels of DNA sequence divergence in some cases, for example, at the COI locus, a maximum of 3.6% among more than 8 species in one sub-clade is observed. This observation in itself suggests recent speciation, which cannot readily be dismissed as taxonomic over-splitting as the fine-scale taxonomic subdivision of *Mecodema* is based on sound morphological and ecological criteria. Interestingly *Mecodema* diversity does not comprise only allopatric populations, which might be expected under a model of recent spatial isolation; many species exist in sympatry with congenerics. Furthermore, *Mecodema* is commonly also found with other carabids (e.g. *Megadromus* (Motschulsky, 1866)), implying additional niche competition [48] resulting in fine scale ecological delimitation. Thus, high species diversity in *Mecodema* might well be the product of intense selection yielding adaptive radiation. A striking example is that of *Metaglymma* (Bates, 1867), which by virtue of its distinct morphology was classified in a separate genus, but is probably better treated as an ecologically specialized *Mecodema*. *Metaglymma* may, along with the coastal monotypic *Brullea*, be included in *Mecodema* following further morphological and genetic investigation. The close relationship between these three genera, compared to the two other mainland New Zealand outgroup genera *Diglymma* and *Oregus*, is consistent with the degree of morphological differences among them [45]. Additional sampling of taxa and populations is necessary to interpret phylogeographic and taxonomic patterns in detail, but the current level of sampling is sufficient for the purposes of gauging the timing of species radiation.

Molecular dating with appropriate calibrations provides an empirical approach to estimate timing of past speciation events and phylogeography [73]. Determining the age of endemic biota has been largely dependent on the use of molecular clock calibrations, and the dating of speciation in New Zealand's plants and animals is especially problematic due to the generally poor fossil record for many lineages. Even where fossils are present, their use requires good time constraint and confidence in their ancestral status for respective extant taxa [73].

Substitution rates of mitochondrial genes differ greatly among genes and lineages and therefore the use of a general invertebrate divergence rates would be inappropriate for this study. Bayesian relaxed clock methods on the other hand allow rates to vary among lineages although accuracy might still be influenced greatly by the setting of priors. In this study the inferred mitochondrial divergence times based on the fast COI rate obtained for Coleoptera [44] are consistently substantially younger than would be expected for a highly diversified genus even in the relatively youthful landscape of New Zealand.

Despite a perception that New Zealand is an ancient land mass with an ancient biota, diversity is increasingly shown to be the product of comparatively recent speciation congruent with Plio-Pleistocene tectonic activity since the mid-Miocene [7], [8], [74]. Speciation of plants (e.g., [75]) and animals including vertebrates (e.g., [76], [77]) often correlates with relatively recent but profound environmental changes and habitat diversification. We conclude that radiation of the *Mecodema* crown group is unlikely to have started before the mid to late Miocene, with most lineage formation most likely in the Pliocene and Pleistocene. This relatively shallow radiation is therefore consistent with the timing of radiations inferred for other New Zealand invertebrates (cicada [43], weta [78], [79], cockroach [80], isopod [81]), vertebrates (galaxiid fish [82], [83], geckos [84]) and plants (buttercup [85], *Pachycladon*, [38], [86]).

On the basis of a conservative rate estimate for molecular evolution most diversification seems to have happened since the Pliocene (>5 Myr). The inference from this is that net diversification (comprising species origination and species extinction) within this genus has been strongly influenced by recent geophysical processes, such as mountain building in the Pliocene, habitat shifting and land formation in the Pleistocene. On a broad scale, and considering the relationships within *Mecodema* and calibrations used, at least eight exchanges between the two main islands of New Zealand can be inferred (Fig. 3). All of the splits between North Island and South Island sister taxa include species now distributed in the mid or lower North Island of New Zealand; areas that were mostly below sea level until recent geological time [28]. Considering the availability of land during this time frame, biotic exchange between the two main islands was most probably from south to north. South Island has been of similar size as it is today, at least since early Pliocene, whereas southern North Island formed only in the last 1 or 2 Myr ([28] and references therein). Prior to this, the proto-North Island was probably smaller than today and at least in the Pliocene was further from northern South Island than it is now. More complex scenarios involving multiple range shifts, speciation events and extinction are possible, but cannot be inferred from the present data.

The splits between sister taxa currently occupying either North or South Island range in estimated age using stratigraphy from 6.39 Myr to 1.60 Myr (Fig. 3A). This suggests numerous exchanges between these regions since the Pliocene involving, most probably, colonisation after over-sea dispersal, rather than in situ radiation from a single ancestor in each island. The dominance of South Island lineages within *Mecodema*, *Metaglymma* and the *Diglymma/Oregus* sister clade are consistent with the notion that colonisation has for the most part been northwards.

The current sampling is not complete in terms of living species or populations. Additional sampling is most likely to reduce the inferred age of formation of species lineages (by dividing existing branches), which could increase the number of inferred dispersal events. Alternatively, detailed population sampling could indicate taxonomic revision is required, and this might lead to slightly older inferred ages of named species. Nevertheless, a number of observations can be made: Geographically proximate species are rarely sister taxa (e.g. *M. costellum*, *M. crenicolle*, *M. strictum* in north east South Island). Some localised species might have arisen via allopatry on past islands, for example *M.* n. sp. in eastern central North Island (light green triangle in Fig. 3). Many populations and species have their range (sampled and known) in areas that were under water during late Pliocene and early Pleistocene (e.g. *M. crenicolle/crenaticolle*, *M. oconnori*, *M. simplex*). The existence of these indicates speciation since land became available and we note a mismatch between estimated lineage age using Chatham stratigraphy and estimates of land age. For example the split within North Island and South Island *M. crenicolle/crenaticolle* complex is estimated at 2.87 Myr. This predates evidence for land in southern North Island by more than 1 Myr, and predates by at least 1.5 Myr the most substantial period of land connection between North Island and South Island in the late Pleistocene. This type of mismatch between dates derived from genetics and those from geology might result from lineages evolving before the extant species they yielded arrived where they occur today as species ranges do change over time. In North Island, New Zealand, molecular studies showing this type of range expansion include tree weta [78] and stick insects [22]. However, evidence of recent range expansion is most easily found when populations of the taxon concerned still exist at source locations as well as colonised locations. In *Mecodema*, range shift after lineage origination would require lineage splitting, persistence of two lineages at a source location, expansion of at least one lineage and extinction of that lineage at the source. An alternative and simpler explanation is that the molecular rates inferred for these data by Chatham stratigraphic calibration are underestimated, which has thus yielded over estimation of lineage age. Such an inference is supported by the much younger times yielded by calibration with the Coleoptera COI rate [44].

It is not possible to determine using the present data when, after emergence of the Chatham Islands, the *Mecodema alternans* lineage arrived there. In all likelihood the age estimates from stratigraphic calibration can be considered maximal. Using this approach, we can infer a rate of molecular evolution for *Mecodema* COI of 0.0059 subst/site/myrs/l [87], which is substantially lower than comparable estimates for Coleoptera that range from 0.02 [65] to 0.086 subst/site/myrs/l [44]. Together with a lack of taxonomic distinction between mainland and Chatham populations this is consistent with a scenario of colonisation late in Chatham Island history. If so, then the entire *Mecodema* radiation is supported as even younger than we are currently able to demonstrate, with a larger proportion of speciation since the late Pliocene.

The fact that this genus and other large flightless insects are present on the Chatham Islands [8], [18] and must have got there over sea supports the plausibility of trans-Tasman colonisation of the ancestral lineage to New Zealand. While the lineage (stem group) in New Zealand might date back to the separation of Zealandia from Gondwanaland having persisted despite marine transgression before the Miocene, it might equally have arrived in New Zealand in the Miocene as additional land became available. Regardless of the timing and mode of origination of *Mecodema* in New Zealand, the more important evolutionary feature of this beetle, with respect to the assembly of the native biota, is diversification of the crown group. Evidently, a few million years have been sufficient for the evolution of a complex ecosystem comprising not simply allopatric subunits but an array of sympatric species with distinct ecologies; for this to happen a long geological history was not required. While it has been predicted that intense phylogeographic structuring and speciation dating to the Plio-Pleistocene might be observed more frequently in naturally subdivided alpine conditions than in lowland forests [88], *Mecodema* speciation appears to provide an example where diversification has proceeded across space and into diverse habitats, from coast to above the tree line. Future work on the detailed ecology of these species will be instrumental in demonstrating the population genetic and ecological mechanisms of diversification (e.g. [89]).

Increasingly, the fields of species phylogenetics and population phylogeography are merging as it becomes easier to generate appropriate DNA data, and the focus in taxonomy is shifted towards an evolutionary paradigm (e.g. [90]. Teasing apart the interaction of abiotic and genetic processes on population subdivision remains challenging but *Mecodema* is one taxon group that will provide helpful insight, and it is already evident that *Mecodema* is an impressive example of recent species radiation in the New Zealand fauna. In recent years, synthesis of phylogenetic, ecological and taxonomic evidence has indicated that the biology of New Zealand is primarily the story of recent adaptation and speciation [8], [9], [19]. Understanding properly how our view of “recent” geological time relates to the intergenerational population genetics of large invertebrate populations will provide the basis of exciting research.

## References

[1] Daugherty CH, Gibbs GW, Hitchmough RA (1993) Mega-island or micro-continent? New Zealand and its fauna. Trends Ecol Evol 8: 437–442. 21236224 10.1016/0169-5347(93)90006-B

[2] Trewick SA, Morgan-Richards M (2009) New Zealand Biology. In Encyclopedia of Islands. (R. G. Gillespie and D. A. Clague, eds.) University of California Press (pp. 665–673).

[3] Campbell HJ, Hutching G (2007) In search of ancient New Zealand. Lower Hutt, New Zealand: Penguin, Auckland & GNS Science.

[4] Trewick SA, Paterson AM, Campbell HJ (2007) Hello New Zealand. J Biogeog 34: 1–6.

[5] Landis CA, Campbell HJ, Beg JG, Mildenhall DC, Paterson AM, et al (2008) The Waipounamu erosion surface: questioning the antiquity of the New Zealand land surface and terrestrial fauna and flora. Geol Mag 145: 1–25.

[6] Fleming CA (1979) The Geological history of New Zealand and its life. Auckland, Auckland University Press.

[7] Cooper RA, Millener PR (1993) The New Zealand biota: Historical background and new research. Trends Ecol Evol 8: 429–433. 21236222 10.1016/0169-5347(93)90004-9

[8] Goldberg J, Trewick SA, Paterson AM (2008) Evolution of New Zealand's terrestrial fauna: a review of molecular evidence. Philos Trans R Soc Lond B Biol Sci 363: 3319–3334. 18782728 10.1098/rstb.2008.0114 PMC2607375

[9] Wallis GP, Trewick SA (2009) New Zealand phylogeography: evolution on a small continent. Mol Ecol 18: 3548–3580. 19674312 10.1111/j.1365-294X.2009.04294.x

[10] Trewick SA, Gibb GC (2010) Vicars, tramps and assembly of the New Zealand avifauna: a review of molecular phylogenetic evidence. Ibis 152: 226–253.

[11] Gillespie RG, Roderick GK (2002) Arthropods on islands: Colonization, speciation, and conservation. Annu Rev Entomol 47: 595–632. 11729086 10.1146/annurev.ento.47.091201.145244

[12] Christidis L, Leeton PR, Westerman M (1996) Were bowerbirds part of the New Zealand fauna? Proc Natl Acad Sci U S A 93: 3898–3901. 8632986 10.1073/pnas.93.9.3898 PMC39456

[13] Winkworth RC, Wagstaff SJ, Glenny D, Lockhart PJ (2002) Plant dispersal N.E.W.S from New Zealand. Trends Ecol Evol 17: 514–520.

[14] De Queiroz A (2005) The resurrection of oceanic dispersal in historical biogeography. Trends Ecol Evol 20: 68–73. 16701345 10.1016/j.tree.2004.11.006

[15] Knapp M, Stöckler K, Havell D, Delsuc F, Sebastiani F, et al (2005) Relaxed molecular clock provides evidence for long-distance dispersal of *Nothofagus* (Southern Beech). PloS Biol 3: e14. 15660155 10.1371/journal.pbio.0030014 PMC539330

[16] Perrie L, Brownsey P (2007) Molecular evidence for long-distance dispersal in the New Zealand pteridophyte flora. J Biogeog 34: 2028–2038.

[17] Chapple DG, Ritchie PA, Daugherty CH (2009) Origin, diversification, and systematics of the New Zealand skink fauna (Reptilia: Scincidae). Mol Phylogenet Evol 52: 470–487. 19345273 10.1016/j.ympev.2009.03.021

[18] Goldberg J, Trewick SA (2011) Exploring phylogeographic congruence in a continental island system. Insects 2: 369–399. 26467734 10.3390/insects2030369 PMC4553550

[19] Pons J, Fujisawa T, Claridge EM, Savill RA, Barraclough TG, et al (2011) Deep mtDNA subdivision within Linnean species in an endemic radiation of tiger beetles from New Zealand (genus *Neocicindela*). Mol Phylogenet Evol 56: 796–807. 10.1016/j.ympev.2011.02.013 21338699

[20] Trewick SA, Wallis GP, Morgan-Richards M (2011) The invertebrate life of New Zealand: a phylogeographic approach. Insects 2: 297–325. 26467729 10.3390/insects2030297 PMC4553545

[21] Marshall DC, Hill KBR, Fontaine KM, Buckley TR, Simon C (2009) Glacial refugia in a maritime temperate climate: Cicada (*Kikihia subalpina*) mtDNA phylogeography in New Zealand. Mol Ecol 18: 1995–2009. 19434813 10.1111/j.1365-294x.2009.04155.x

[22] Morgan-Richards M, Trewick SA, Stringer IAN (2010) Geographic parthenogenesis and the common tea-tree stick insect of New Zealand. Mol Ecol 19: 1227–1238. 20163549 10.1111/j.1365-294X.2010.04542.x

[23] Marske KA, Leschen RAB, Buckley TR (2011) Reconciling phylogeography and ecological niche models for New Zealand beetles: Looking beyond glacial refugia. Mol Phylogenet Evol 59: 89–102. 21262367 10.1016/j.ympev.2011.01.005

[24] Chappell EM, Trewick SA, Morgan-Richards M (2012) Shape and sound reveal genetic cohesion not speciation in the New Zealand orthopteran, *Hemiandrus pallitarsis*, despite high mtDNA divergence. Biol J Linn Soc 105: 169–186.

[25] Marshall DC, Slon K, Cooler JR, Hill KBR, Simon C (2008) Steady Plio-Pleistocene diversification and a 2-million-year sympatry threshold in a New Zealand cicada radiation. Mol Phylogenet Evol 48: 1054–1066. 18590969 10.1016/j.ympev.2008.05.007

[26] Cooper A, Cooper RA (1995) The Oligocene bottleneck and New Zealand biota: Genetic record of a past environmental crisis. Proc Biol Sci B 261: 293–302. 10.1098/rspb.1995.0150 8587872

[27] Kamp PJJ (1992) Tectonic architecture of New Zealand. Landforms of New Zealand. 2^nd^ ed. Longman Paul Ltd. New Zealand. pp 1–31.

[28] Trewick SA, Bland KJ (2012) Fire and slice: Paleaogeography for biogeography at New Zealand's North Island/South Island juncture. J R Soc N Z 42: 153–183.

[29] Lee DE, Lee WG, Mortimer N (2001) Where and why have all the flowers gone? Depletion and turnover in the New Zealand Cenozoic angiosperm flora in relation to palaeogeography and climate. Aust J Bot 49: 341–356.

[30] Worthy TH, Tennyson AJD, Jones C, McNamara JA, Douglas BJ (2007) Miocene waterfowl and other birds from central Otago, New Zealand. J Syst Palaeontol 5: 1–39.

[31] Landis CA, Campbell HJ, Begg JG, Mildenhall DC, Paterson AM, et al (2010) Discussion of ‘The Waipounamu erosion surface: questioning the antiquity of the New Zealand land surface and terrestrial fauna and flora’. Geol Mag 147: 151–155.

[32] Campbell HJ (1998) Fauna and flora of the Chatham Islands: Less than 4 MY old? Geological Society of New Zealand Miscellaneous Publications 97: 15–16.

[33] Campbell HJ, Begg JG, Beu AG, Carter RM, Davies G, et al (2006) On the turn of a scallop. Geological Society of New Zealand Miscellaneous Publications 121: 9.

[34] Campbell HJ, Begg JG, Beu AG, Carter RM, Curtis N, et al (2009) Geological considerations relating to the Chatham Islands, mainland New Zealand and the history of New Zealand terrestrial life. Geological Society of New Zealand Miscellaneous Publications 126: 5–6.

[35] Trewick SA (2000) Molecular evidence for dispersal rather than vicariance as the origin of flightless insect species on the Chatham Islands. New Zealand. J Biogeog 27: 1189–1200.

[36] Wagstaff SJ, Garnock-Jones PJ (1998) Evolution and biogeography of the *Hebe* complex (Scrophulariaceae) inferred from ITS sequences. NZ J Bot 36: 425–437.

[37] Shepherd LD, de Lange PJ, Perrie LR (2009) Multiple colonizations of a remote oceanic archipelago by one species: how common is long-distance dispersal? J Biogeog 36: 1972–1977.

[38] Heenan PB, Mitchell AD, de Lange PJ, Keeling J, Paterson AM (2010) Late-Cenozoic origin and diversification of Chatham Islands endemic plant species revealed by analyses of DNA sequence data. NZ J Bot 48: 83–136.

[39] Boon WM, Kearvell JC, Daugherty CH, Chambers GK (2001) Molecular systematics and conservation of Kakariki (*Cyanoramphus* spp.). Science for Conservation (Wellington, N.Z.) 176.

[40] Millener PR, Powlesland RG (2001) The Chatham Islands pigeon (Parea) deserves full species status; *Hemiphaga chathamensis* (Rothschild 1891); Aves: Columbidae. J R Soc N Z 31: 365–383.

[41] Goldberg J, Trewick SA, Powlesland RG (2011) Population structure and biogeography of *Hemiphaga* pigeons (Aves: Columbidae) on islands in the New Zealand region. J Biogeog 38: 285–298.

[42] Arensburger P, Simon C, Holsinger K (2004) Evolution and phylogeny of the New Zealand cicada genus *Kikihia* Dugdale (Homoptera: Auchenorrhyncha: Cicadidae) with special reference to the origin of the Kermadec and Norfolk Islands species. J Biogeog 31: 1769–1783.

[43] Paterson AM, Trewick SA, Armstrong K, Goldberg J, Mitchell A (2006) Recent and emergent: molecular analysis of the biota supports a young Chatham Islands. Geological Society of New Zealand Miscellaneous Publications 121: 27–29.

[44] Pons J, Ribera I, Bertranpetit J, Balke M (2010) Nucleotide substitution rates for the full set of mitochondrial protein-coding genes in Coleoptera. Mol Phylogenet Evol 56: 796–807. 20152911 10.1016/j.ympev.2010.02.007

[45] Roig-Juňent S (2000) The subtribes and genera of the tribe Broscini (Coleoptera: Carabidae): Cladistic analysis, taxonomic treatment, and biogeographical consideration. Bull Am Mus Nat Hist 255.

[46] Britton EB (1949) The Carabidae (Coleoptera) of New Zealand, Part III – A revision of the tribe Broscini. Trans R Soc N Z 77: 533–581.

[47] Larochelle A, Larivière M-C (2001) Carabidae (Insecta: Coleoptera): catalogue. Fauna of New Zealand, Number 43 (pp. 44–61). Lincoln, Canterbury, New Zealand, Manaaki Whenua Press.

[48] Hutchison MAS (2001) Habitat use, seasonality and ecology of carabid beetles (Coleoptera: Carabidae) in native forest remnants, North Island, New Zealand. Master thesis, Palmerston North, Massey University, New Zealand.

[49] Townsend JI (1971) Entomology of the Aucklands and other islands south of New Zealand: Coleoptera: Carabidae: Broscini. Pacific Island Monographs 27: 178–180.

[50] Pawson SM, Emberson RM, Armstrong KF, Paterson AM (2003) Phylogenetic revision of the endemic New Zealand carabid genus *Oregus* Putzeys (Coleoptera : Carabidae : Broscini). Invertebr Syst 17: 625–640.

[51] Ruiz C, Jordal BH, Emerson BC, Will KW, Serrano J (2010) Molecular phylogeny and Holarctic diversification of the subtribe Calathina (Coleoptera: Carabidae: Sphodrini. Mol Phylogenet Evol 55: 358–371. 19900569 10.1016/j.ympev.2009.10.026

[52] Maddison DR, Baker MD, Ober KA (1999) Phylogeny of carabid beetles as inferred from 18S ribosomal DNA (Coleoptera: Carabidae). Syst Ent 24: 103–138.

[53] Sunnucks P, Hales DF (1996) Numerous transposed sequences of mitochondrial Cytochrome Oxidase I–II in aphids of the genus *Sitobion* (Hemiptera: Aphididae). Mol Biol Evol 13: 510–524. 8742640 10.1093/oxfordjournals.molbev.a025612

[54] Trewick S (2008) DNA Barcoding is not enough: mismatch of taxonomy and genealogy in New Zealand grasshoppers (Orthoptera: Acrididae). Cladistics 24: 240–254.

[55] Willerslev E, Cooper A (2005) Ancient DNA. Proc Biol Sci B 272: 3–16. 10.1098/rspb.2004.2813 PMC1634942 15875564

[56] Simon C, Frati F, Beckenbach A, Crespi B, Liu H, et al (1994) Evolution, weighting and phylogenetic utility of mitochondrial gene sequences and a compilation of conserved polymerase chain reaction primers. Ann Entomol Soc Am 87: 651–701.

[57] Vawter L (1991) Evolution of blattoid insects and of the small subunit ribosomal gene. PhD dissertation, University of Michigan, Ann Arbor.

[58] Rambaut A (1996) Se-Al: Sequence Alignment Editor. (Available: http://tree.bio.ed.ac.uk/software/seal/Accessed 2013 October)

[59] Ronquist F, Huelsenbeck JP (2003) MrBayes3: Bayesian phylogenetic inference under mixed models. Bioinformatics 19: 1572–1574. 12912839 10.1093/bioinformatics/btg180

[60] Farris JS, Källersjö M, Kluge AG, Bult C (1994) Testing significance of incongruence. Cladistics 10: 315–319.

[61] Swofford DL (1998) PAUP*: Phylogenetic analysis using Parsimony (*and other methods) Version 4. Sinauer Associates, Sunderland, MA.

[62] Stematakis A (2006) RAxML-VI-HPC: maximum likelihood-based phylogenetic analyses with thousands of taxa and mixed models. Bioinformatics 22: 2688–2690. 16928733 10.1093/bioinformatics/btl446

[63] Miller MA, Pfeiffer W, Schwartz T (2010) Creating the CIPRES Science Gateway for inference of large phylogenetic trees, pp. 1–8. New Orleans, LA, Proceedings of the Gateway Computing Environments Workshop (GCE).

[64] Pons J, Vogler AP (2005) Complex pattern of coalescence and fast evolution of a mitochondrial rRNA pseudogene in a recent radiation of Tiger Beetles. Mol Biol Evol 22: 991–1000. 15647517 10.1093/molbev/msi085

[65] Ribera I, Fresneda J, Bucur R, Izquierdo A, Vogler AP, Salgado JM, Cieslak A (2010) Ancient origin of a Western Mediterranean radiation of subterranean beetles. BMC Evol Biol 10: 29. 20109178 10.1186/1471-2148-10-29 PMC2834687

[66] Brower AVZ (1994) Rapid morphological radiation and convergence among races of the butterfly *Heliconius erato* inferred from patterns of mitochondrial DNA evolution. Proc Natl Acad Sci U S A 91: 6491–6495. 8022810 10.1073/pnas.91.14.6491 PMC44228

[67] Drummond AJ, Ho SYW, Phillips MJ, Rambaut A (2006) Relaxed phylogenetics and dating with confidence. PLoS Biol 4: e88. 16683862 10.1371/journal.pbio.0040088 PMC1395354

[68] Kass RE, Raftery AE (1995) Bayes Factors. J American Statist Assoc 90: 773–795.

[69] Drummond AJ, Rambaut A (2007) BEAST: Bayesian evolutionary analysis by sampling trees. BMC Evol Biol 7: 214. 17996036 10.1186/1471-2148-7-214 PMC2247476

[70] Drummond AJ, Suchard MA, Xie D, Rambaut A (2012) Bayesian phylogenetics with BEAUti and the BEAST 1.7. Mol Biol Evol 29: 1969–1973. 22367748 10.1093/molbev/mss075 PMC3408070

[71] Rambaut A, Drummond AJ (2007) Tracer v1.4. (Available: http://beast.bio.ed.ac.uk/Tracer. Accessed 2013 August)

[72] Posada D, Crandall KA (1998) Modeltest: Testing the model of DNA substitution. Bioinformatics 14: 817–818. 9918953 10.1093/bioinformatics/14.9.817

[73] Ho SYW, Phillips MJ (2009) Accounting for calibration uncertainty in phylogenetic estimation of evolutionary divergence times. Syst Biol 58: 367–380. 20525591 10.1093/sysbio/syp035

[74] Cande SC, Stock JM (2004) Pacific-Antarctic-Australia motion and the formation of the Macquarie Plate. Geophysical Journal International 157: 399–414.

[75] Meudt HM, Simpson BB (2006) The biogeography of the austral, subalpine genus *Ourisia* (Plantaginaceae) based on molecular phylogenetic evidence: South American origin and dispersal to New Zealand and Tasmania. Biol J Linn Soc Lond 87: 479–513.

[76] Baker AJ, Huynen LJ, Haddrath O, Millar CD, Lambert DM (2005) Reconstructing the tempo and mode of evolution in an extinct clade of birds with ancient DNA: the giant moas of New Zealand. Proc Natl Acad Sci U S A 102: 8257–8262. 15928096 10.1073/pnas.0409435102 PMC1149408

[77] Bunce M, Szulkin M, Lerner HRL, Barnes I, Shapiro B, et al (2005) Ancient DNA provides new insights into the evolutionary history of New Zealand's extinct Giant Eagle. PLoS Biol 3: 44–46. 10.1371/journal.pbio.0030009 PMC539324 15660162

[78] Morgan-Richards M, Trewick SA, Wallis G (2001) Chromosome races with Pliocene origins: Evidence from mtDNA. Heredity 86: 303–312. 11488967 10.1046/j.1365-2540.2001.00828.x

[79] Trewick SA, Morgan-Richards M (2005) After the deluge: mitochondrial DNA indicates Miocene radiation and Pliocene adaptation of tree and giant weta (Orthoptera: Anostostomatidae). J Biogeog 32: 295–309.

[80] Chinn WG, Gemmell NJ (2004) Adaptive radiation within New Zealand endemic species of the cockroach genus *Celatoblatta* Johns (Blattidae): A response to Plio-Pleistocene mountain building and climate change. Mol Ecol 13: 1507–1518. 15140094 10.1111/j.1365-294X.2004.02160.x

[81] McGaughran A, Hogg ID, Stevens MI, Chadderton WL, Winterbourn MJ (2006) Genetic divergence of three freshwater isopod species from southern New Zealand. J Biogeog 33: 23–30.

[82] Waters JM, Wallis GP (2001) Mitochondrial DNA phylogenetics of the *Galaxias vulgaris* complex from South Island, New Zealand: rapid radiation of a species flock. J Fish Biol 58: 1166–1180.

[83] Burridge CP, Craw D, Fletcher D, Waters JM (2008) Geological dates and molecular rates: Fish DNA sheds light on time dependency. Mol Biol Evol 25: 624–633. 18281273 10.1093/molbev/msm271

[84] Nielsen SV, Bauer AM, Jackmann TR, Hitchmough RA, Daugherty CH (2011) New Zealand geckos (Diplodactylidae): Cryptic diversity in a post-Gondwanan lineage with trans-Tasman affinities. Mol Phylogenet Evol 59: 1–22. 21184833 10.1016/j.ympev.2010.12.007

[85] Lockhart PJ, McLenachan PA, Havell D, Glenny D, Huson D, et al (2001) Phylogeny, radiation, and transoceanic dispersal of New Zealand alpine buttercups: Molecular evidence under split decomposition. Ann Mo Bot Gard 88: 458–477.

[86] Heenan PB, Mitchell AD (2003) Phylogeny, biogeography and adaptive radiation of *Pachycladon* (Brassicaceae) in the mountains of South Island, New Zealand. J Biogeog 30: 1737–1749.

[87] Goldberg J (2010) Speciation and phylogeography in the New Zealand archipelago. PhD dissertation, Massey University, Palmerston North, New Zealand

[88] Trewick SA, Wallis GP, Morgan-Richards M (2000) Phylogeographical pattern correlates with Pliocene mountain building in the alpine scree weta (Orthoptera, Anostostomatidae). Mol Ecol 9: 657–666. 10849282 10.1046/j.1365-294x.2000.00905.x

[89] Sota T, Ishikawa R (2004) Phylogeny and life-history evolution in Carabus (subtribe Carabina: Coleoptera, Carabidae) based on sequences of two nuclear genes. Biol J Linn Soc Lond 81: 135–149.

[90] Marshall DC, Hill KBR, Colley JR, Simon C (2011) Hybridization, mitochondrial DNA phylogeography, and prediction of the early stages of reproductive isolation: lessons from New Zealand cicada (Genus *Kikihia*). Syst Biol 60: 482–502. 21471306 10.1093/sysbio/syr017

